# Hepatosteatosis from Lysosomal Acid Lipase Deficiency

**DOI:** 10.1007/s11605-018-3906-7

**Published:** 2018-08-06

**Authors:** Stephan Zandanell, Florian Primavesi, Elmar Aigner

**Affiliations:** 1 First Department of Medicine, Paracelsus Medical University Salzburg, Müllner Hauptstrasse 48, 5020 Salzburg, Austria; 2 Department of Visceral, Transplant and Thoracic Surgery, Medical University of Innsbruck, Innsbruck, Austria

**Keywords:** LAL deficiency, Lysosomal acid lipase, NAFLD, Steatosis, Herniotomy, Laparoscopy

## History and Clinical Course

**Fig. 1:**
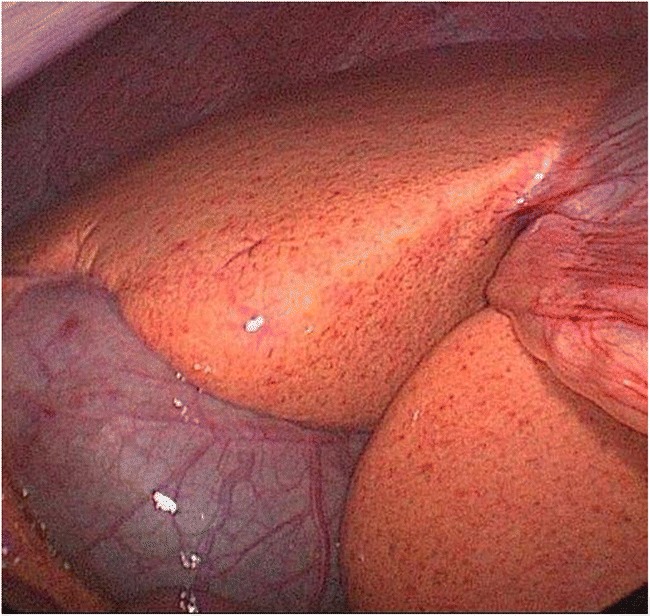
Laparoscopic view showing orange-colored liver surface

**Fig. 2:**
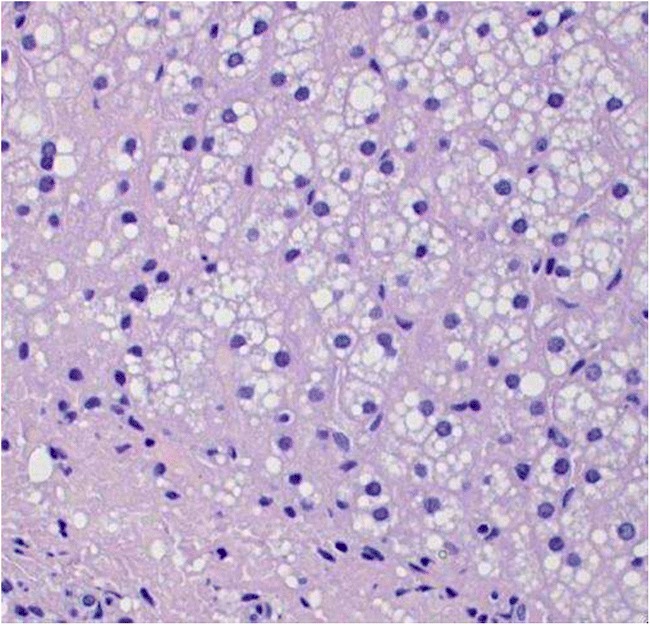
Microvesicular steatosis with extensive fibrosis

A 21-year-old clinically well male (BMI 20.8 kg/m^2^) with unremarkable family and medical history presented to the surgical outpatient clinic with a two-day history of left side inguinal pain and was diagnosed with uncomplicated hernia. Transabdominal preperitoneal patch repair was scheduled and performed several weeks later. During herniotomy, a “massive liver steatosis with completely orange surface” was reported by the surgeon and liver biopsy specimens were obtained (Fig. 1). Pathological examination reported 50–60% microvesicular steatosis affecting hepatocytes and Kupffer cells with portal, periportal, and septal fibrosis (Fig. 2). Upon referral to the hepatologist, biochemical work-up revealed total cholesterol 265 mg/dL, LDL 211 mg/dL, HDL 39 mg/dL, triglycerides 164 mg/dL, ALT 56 IU/L, and a serum ferritin of 277 μg/L with otherwise normal laboratory reports. Infectious, autoimmune, or metabolic liver diseases such as Wilson’s disease were ruled out. The patient did not use and had never used any medication or illicit drugs and consumed moderate amounts of alcohol on weekends.

## Diagnosis

Due to the suggestive histological picture with exclusively microvesicular steatosis, a test for activity of lysosomal acid lipase (LAL) was performed at the visit at the hepatology outpatient clinic and LAL activity was reported to be 0.0 nmol/3 h (normal 0.1–2.0 nmol/3 h) which is diagnostic of LAL deficiency (LAL-D). A homozygous mutation of E8SJM (c.894G>A) was confirmed. LAL-D is a rare and under-diagnosed autosomal-recessive lysosomal storage disease. Complete lack of LAL activity, traditionally known as Wolman disease, is lethal within the first year of life due to liver failure and malabsorption. Minimal residual LAL activity is referred to as cholesterol ester storage disease (CESD) and typically manifests in childhood or early adulthood with microvesicular hepatic steatosis progressing to fibrosis, cirrhosis, and potentially hepatocellular carcinoma.^1^ Orange coloration of steatosis is a result of predominant cholesterol deposition and different from the yellow appearance ensuing from triglyceride accumulation associated with more common fatty liver diagnoses such as nonalcoholic or alcoholic fatty liver disease. Hallmark laboratory abnormalities include high LDL, total cholesterol, and triglycerides which are mainly attributed to activation of de novo lipogenesis via SREBP1c as a result of low cytoplasmatic concentration of free cholesterol and fatty acids.^2^ Low HDL is a consequence of decreased activity of LXR-dependent HDL production.^2^ Children or adults with LAL-D usually progress to end-stage liver disease with its complications early in life and are at high risk of developing premature atherosclerosis with cerebral or myocardial infarction. It is crucial to raise awareness of this rare disease as specific enzyme replacement therapy with sebelipase alfa has become available and timely diagnosis may therefore be life-saving for affected subjects.^3^
